# Selective extraction of aliphatic amines by functionalized mesoporous silica-coated solid phase microextraction Arrow

**DOI:** 10.1007/s00604-019-3523-5

**Published:** 2019-06-11

**Authors:** Hangzhen Lan, Wenzhong Zhang, Jan-Henrik Smått, Risto T. Koivula, Kari Hartonen, Marja-Liisa Riekkola

**Affiliations:** 1 Department of Chemistry, University of Helsinki, P.O. Box 55, 00014 Helsinki, Finland; 2 Institute for Atmospheric and Earth System Research, University of Helsinki, P.O. Box 64, 00014 Helsinki, Finland; 3 Laboratory of Physical Chemistry, Åbo Akademi University, Porthansgatan 3-5, 20500 Turku, Finland

**Keywords:** Functionalized mesoporous silica material, Solid phase microextraction Arrow, Selective extraction, Low-molecular-weight aliphatic amines, Gas chromatography-mass spectrometry, Mushroom, Atmospheric air, Urine

## Abstract

Mesoporous silica-coated solid phase microextraction (SPME) Arrow systems were developed for capturing of low-molecular-weight aliphatic amines (LMWAAs) from complicated sample matrices. Specifically, silicas of type MCM-41, SBA-15 and KIT-6 were chosen as substrates to afford size-exclusion selectivity. They possess ordered multidimensional pore-channel structures and mesopore sizes between 3.8 and 8.2 nm. Their surface acidity was enhanced by grafting them with a layer of titanium hydrogenphosphate (-TP). This enhanced the chemical selectivity for basic LMWAAs. The siliceous coatings increased the extraction of ethylamine, diethylamine (DEA) and triethylamine (TEA) by factors of 18.6–102.5, 4.8–10.8 and 2.6–4.0, respectively, when compared to the commercial SPME Arrow with polydimethylsiloxane/divinylbenzene coating. Among them, the MCM-41 and MCM-41-TP coated SPME Arrows demonstrated exceptional selectivity towards LMWAAs that were quantified by gas chromatography-mass spectrometry (GC-MS). The total peak area ratios of LMWAAs/ten competing compounds were 25.4 and 36.3, respectively. The extraction equilibrium was reached within 20–30 min. The MCM-41 and MCM-41-TP derived SPME Arrows gave very similar results (18.4 ± 2.1–376 ± 12 ng g^−1^ to DEA and TEA) when applied to urban mushroom samples. SPME Arrow with MCM-41 coatings followed by GC-MS was applied also to the analysis of atmospheric air and urine samples resulting in high selectivity due to the size and mesoporous structure of the functionalized silica, and its chemical interactions with the LMWAAs.

**Electronic supplementary material:**

The online version of this article (10.1007/s00604-019-3523-5) contains supplementary material, which is available to authorized users.

**Graphical abstract:**
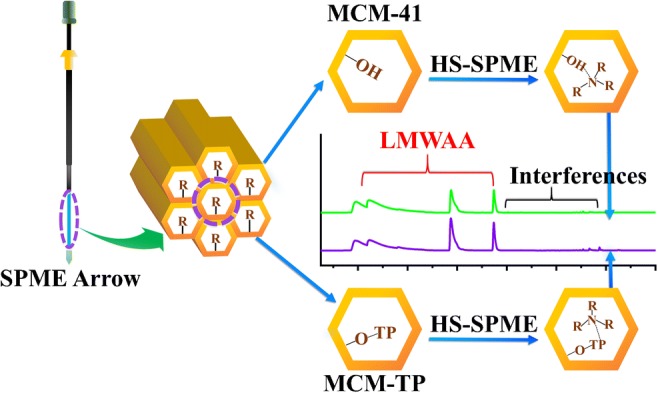
Scheme of synthesis of the MCM-41 silicas, and the preparation of solid phase microextraction Arrow coatings. They were employed for selective capturing of aliphatic amines from complex sample matrices, followed by gas chromatography-mass spectrometry.

## Introduction

Low-molecular-weight C_1_-C_6_ aliphatic amines (LMWAAs) play a vital role in atmospheric and physiological processes. In the Earth’s atmosphere, LMWAAs lead to the formation of salt particles and nitrogen-containing organics and thereby eventually contribute to the nucleation and growth of secondary organic aerosols (SOAs) [1–3]. Natural vegetation constitutes one of the most widespread biosphere sources of the airborne amines [1, 4, 5]. Quantitation of LMWAAs emission from remote regions is therefore essential to help underpin the mechanisms of aerosol formation and growth process. In physiological processes, the breakdown of foodborne choline and lecithin by intestinal microbes produces endogenous LMWAAs [6–8]. However, disruptions on normal hepatic or renal activities cause LMWAAs accumulation in the bloodstream [6, 9], elevating the risk of forming carcinogens (i.e. nitrosamines) and neurotoxins (diethylamine (DEA)) [7, 9, 10]. Hence, early-stage disease diagnosis aided by metabolite profiling benefits from the accurate measurement of urinary LMWAAs concentration.

The measurement of low-concentration (< ng mL^−1^) LMWAAs from complex matrices is not a straightforward task. They are intrinsically highly volatile, polar, reactive, and water soluble. Therefore, their selective and rapid adsorption and enrichment is necessary prior to instrumental analysis. Solid phase microextraction (SPME) Arrow is a simple, time-efficient, robust and reliable sample pretreatment technique that has become more popular just in the past few years [11–15]. In order to achieve targeted separation of LMWAAs, the adsorbent hosted on SPME Arrow needs to be tailored.

Engineered porous materials are widely employed for selective gas adsorption and separation. The selectivity arises synergistically from the synthetic control over the pore-channel systems as well as the surface chemical properties. For gas molecules, micro- and mesoporous materials provide excellent shape recognition. To this end, metal organic frameworks (MOFs), porous carbon-based materials and ordered mesoporous silica, have been designed and used as selective adsorbents due to their tailorable structure, tunable pore geometry, high specific surface area and good porosity [16, 17]. In our previous work, although mesoporous acidified zeolite imidazolate framework-8 (ZIF-8) and microporous Carboxen1000 coated SPME Arrows demonstrated satisfactory extraction selectivity towards LMWAAs in atmospheric air, wastewater, salmon and mushroom samples [12, 14], MOFs had a limited pore accessibility for analytes [14]. Porous carbon-based materials feature an ultrahigh thermal and chemical stability, while difficulties are met in their surface functionalization [18–21]. Mesoporous silica materials offer more flexibility for pore sizes (from a few up to dozens of nanometers) [22] and they have better chemical and thermal stability than MOFs. Further, it is easier to functionalize their mesoporous inner walls compared to those of carbon materials [22]. However, the most promising characteristic of mesoporous silica is its natural acidic surface that predicts a high affinity to basic amines. For all abovementioned merits, ordered mesoporous silica seems to be a good alternative to other classical adsorbents for LMWAAs extraction. Additionally, chemical modification of the mesoporous silica with appropriate functional groups can improve their extraction selectivity towards LMWAAs.

The purpose of this study was to construct mesoporous silica materials for targeted and fast extraction of LMWAAs from different sample matrices. Six ordered mesoporous silica materials with various pore sizes, pore structures and surface functionalities were synthesized and then individually fabricated on SPME Arrow system. Extraction selectivity of the mesoporous silica materials for LMWAAs was clarified, at under 1% and 50% humidity levels, and compared with that of commercial adsorbents. The applicability of SPME Arrow system including our new materials was further tested by exploiting them for detection of LMWAAs in mushroom, atmospheric air and urine samples.

## Experimental

### Reagents and materials

Triethylamine (TEA) (99%), aniline (>99.5%), dimethylformamide (DMF) (99.9%) and polyacrylonitrile (PAN) (Mw = 150,000) were purchased from Sigma-Aldrich (St. Louis, USA); ethanol (100%) and toluene (HPLC grade) were from VWR Chemicals (Pennsylvania, USA); ethylamine (EA) (70% in water), benzyl alcohol (99.5%), diethylamine (DEA) (≥99.7%), benzyl acetate (≥99%) and decane (>99%) were from Fluka (The Netherlands); acetone (≥99.8%), tetrahydrofuran (≥99.9%) and ethyl acetate (≥99.7%) were from Honeywell (Honeywell GmbH, Seelze, Germany); 2-pentylfuran (98%) was from Alfa Aesar (Karlsruhe, Germany); and acetophenone was from The British Drug Houses Ltd. (Poole, England).

Tetraethyl orthosilicate (98%), hexadecyltrimethylammonium bromide (>99.0%), poly(ethylene oxide)-*block*-poly(propylene oxide)-*block*-poly(ethylene oxide) with an average molecular weight of 5800, and 1-butanol (>99.7%) were used for silica synthesis. Titanium (IV) isopropoxide (Ti(OPr^*i*^)_4_) (>97%) and phosphorus oxychloride (>99%) were used for functionalization. All of them were from Sigma-Aldrich.

Polydimethylsiloxane (PDMS), PDMS-Carboxen1000, PDMS-divinylbenzene (DVB) and Carboxen WR SPME Arrows (sorbent film thickness 120 μm and the sorbent length 20 mm) and PAL RTC auto-sampler were kindly provided from CTC Analytics AG (Zwingen, Switzerland). Bare Arrows (for coating length of 20 mm) were from BGB Analytik AG (Zurich, Switzerland).

### Materials synthesis

**Fig. 1:**
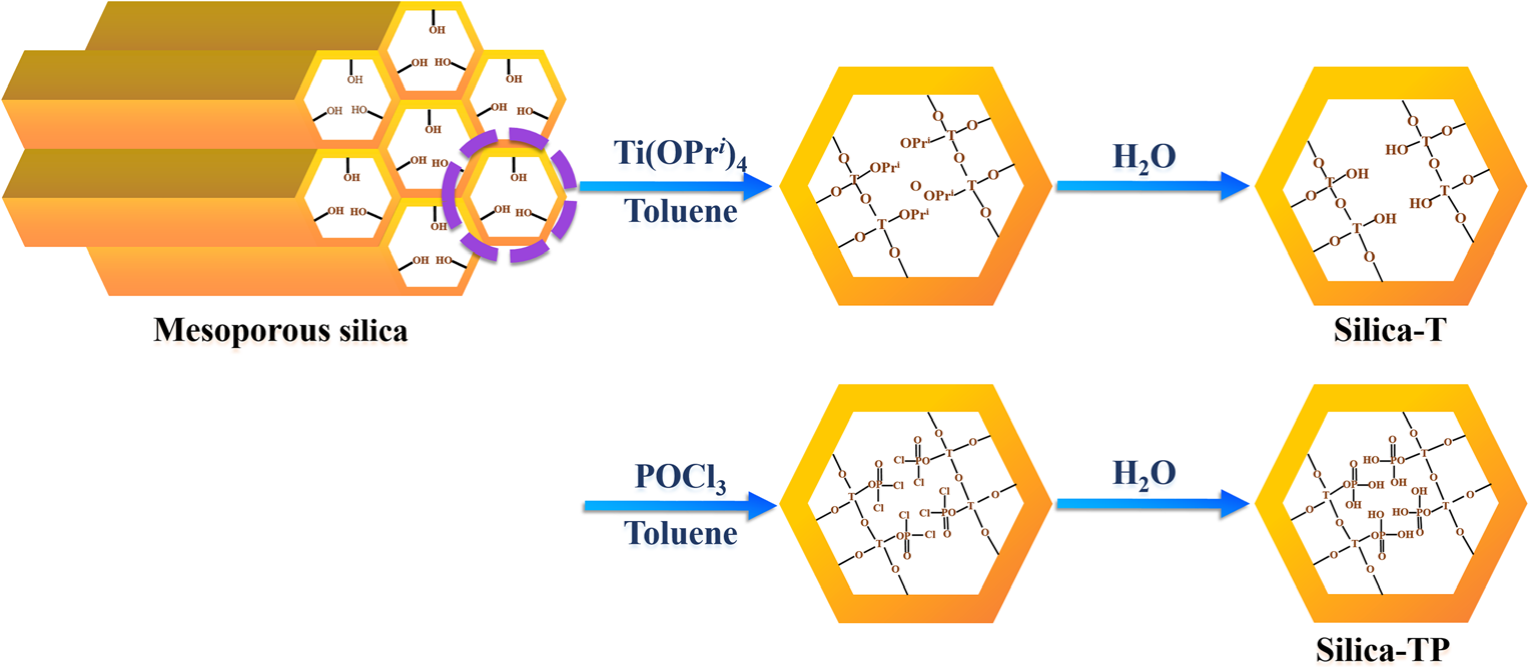
Synthesis route of functionalized silica materials including MCM-TP, SBA-TP and KIT-TP

The mesoporous MCM-41, SBA-15 and KIT-6 silica materials were synthesized via sol-gel template routes following the literature procedures (Fig. 1) [23–25]. The details are given in the Supporting Material.

The functionalization of the mesoporous silica with titanium phosphate moieties was conducted by surface grafting techniques [26]. The details are found from the Supporting Material.

Thus, totally six mesoporous silica materials were synthesized: 1) MCM-41, 2) SBA-15, 3) KIT-6, 4) titanium-phosphate modified MCM-41 (MCM-TP), 5) titanium-phosphate modified SBA-15 (SBA-TP), and 6) titanium-phosphate modified KIT-6 (KIT-TP).

### Fabrication of SPME Arrow coatings

Firstly, 0.2 g of PAN was heated at 90 °C in 10 mL of DMF for 1 h to form the binder solution. After cooling down to room temperature, 0.4 g of adsorbent was added and stirred at 1000 rpm for 18 h. The higher ratio of PAN (0.4 g of PAN+0.4 g of adsorbent) in 10 mL of DMF solution increased viscosity and resulted in a rugged coating. The lower ratio of PAN (0.1 g of PAN+0.4 g of adsorbent) in 10 mL of DMF solution caused the low viscosity and need for more dipping cycles that lengthened the time needed for a satisfactory coating thickness.

Bare SPME Arrows were sequentially cleaned with methanol/water (50/50, *v*/v) and MilliQ water (Millipore DirectQ-UV, Billerica, MA, USA) under ultra-sonication and dried under nitrogen flow.

The cleaned SPME Arrow was gently dipped into the adsorbent/PAN mixture with a total of 12 cycles and dried with nitrogen flow between each cycle. The SPME Arrows were aged in the GC inlet at 250 °C overnight to remove any possible residuals. At the end, six SPME Arrows, coated with MCM-41-, MCM-TP-, SBA-15-, SBA-TP-, KIT-6-, and KIT-TP, were fabricated.

### Diffusion systems for on-line dynamic SPME Arrow

A self-made diffusion system was utilized in this study for constant analyte/gas mixture flow formation. Sample vials including chemical standards were individually placed into a metal cylinder and kept at 30 °C in an oven. A 100 mL min^−1^ of nitrogen flow flushed the cylinder and was mixed with vaporized analytes. Another gas flow (1 L min^−1^), either dry (<1% humidity) or humidified (50% humidity) nitrogen, diluted the mixture afterwards. The confluent gas flow then reached the sampling port used by SPME Arrow. All the selectivity experiments were processed in this mode.

### On-line dynamic SPME Arrow procedures for coating comparison

A PAL Cycle Composer (CTC analytics) software and a PAL RTC auto-sampler controlled and performed all the SPME Arrow steps. Six laboratory-made and four commercial SPME Arrow coatings (PDMS, PDMS-DVB, PDMS-Carboxen1000 and Carboxen WR) were compared in terms of extraction affinity and selectivity towards LMWAAs in dynamic SPME mode. The procedure was as follows: pre-cleaning at 250 °C in the auto-sampler conditioner for 10 min; 10 min extraction in the sampling port at room temperature (~22 °C); desorption at 250 °C in the GC inlet for 1 min.

### SPME Arrow procedures for analytical method development and natural sample analysis

SPME Arrows coated successfully with MCM-41- and MCM-TP were ready for the testing. To a 20 mL headspace vial, 5 mL of sample with 2 g of NaCl were placed and vial was closed with a PTFE/silicone septum screw-cap (both from Phenomenex, Torrance, California, USA). Then 250 μL of 5 M KOH were injected through the septum with a syringe to release the amines into the headspace. The vial was incubated at 40 °C for 15 min. At the same time, SPME Arrow was pre-cleaned at 260 °C for 10 min. Samples were extracted at 40 °C with 250 rpm agitation for 20 min (MCM-41-SPME Arrow) or 30 min (MCM-TP-SPME Arrow). At the end, SPME Arrows were cleaned at 260 °C for 1 min.

### Natural sample preparation

#### Mushroom sample preparation

Six types of mushrooms (Figure S1) were collected from a forest near Kumpula Campus of the University of Helsinki (Helsinki, Finland) on the 8th of October, 2018. They were pretreated and analyzed during the same day without storage. Each sample (4 g) was mixed with 10 mL of 10% formic acid (*v*/v) and homogenized with a kitchen blender (Bosch, Gerlingen, Germany) for 10 min. The homogenate was centrifuged three times at 5000×*g* for 15 min. The supernatants were combined and adjusted to the final volume of 100 mL with 10% formic acid (v/v).

#### Atmospheric air sample preparation

Atmospheric air samples were collected at SMEAR II (Station for Measuring Ecosystem-Atmosphere Relations II) Station in a Scots pine forest at Hyytiälä in southern Finland [27]. The sampling site was located about 15 m from a 127-m high mast for atmospheric and flux measurements (61°50′50.55″N, 24°17′39.77″E, 181 m above the sea level). The forest in Hyytiälä is around 50 years old and dominated by Scots pine, Norway spruce, birch and European aspen. The predominant plant species at the ground level are lingonberry, bilberry, wavy harigrass and heather and the most common mosses are Schreber’s big red stem moss and a dicranum moss [4].

The sampling was done from the 23rd to 26th of October, 2018. The Denuder sampler for air located at 2 m above the ground vegetation and 3 m from the closest tree. A three-channel annular Denuder (242 mm length, Teflon-coated, stainless steel sheath, URG, Chapel Hill, USA) coated with 0.1 M phosphoric acid was connected to a peristaltic pump with a flow rate of 1 m^3^ h^−1^. The sampling times ranged from 4 to 13.5 h. After sampling, the Denuder was rinsed three times with 5 mL of Milli-Q water and the eluent was stored at 4 °C for further analysis. Totally 12 samples were collected.

#### Urine sample

Urine sample was collected from a young male volunteer (28 years old) without additional pretreatment.

### Instrumentation and GC-MS analysis

An Agilent 6890 N gas chromatograph coupled with an Agilent 5975C mass selective detector (Agilent Technologies, Palo Alto, USA) was employed for the GC-MS analysis. An InertCap™ for Amines capillary column (30 m length with 0.25 mm i.d., GL Sciences, Tokyo, Japan) was used for the chromatographic separations. The GC-MS conditions for analysis were as follow: oven temperature program: 40 °C (held for 2 min) and then increased to 250 °C at a rate of 20 °C min^−1^ (held for 5 min). Injector, transfer line, ion source and quadrupole temperature were 260, 250, 230 and 150 °C; respectively. Electron ionization (EI) mode (70 eV) was utilized (*m/z*, 20–350). Helium (99.996%, AGA, Espoo, Finland) was used as carrier gas at a constant flow rate of 1.2 mL min^−1^.

The surface morphologies of mesoporous silica materials were studied by scanning electron microscopy (SEM) (Hitachi, model S-4800, Japan). For their thermal stability measurements under nitrogen flow the thermogravimetric analysis (TGA) with a Mettler Toledo Star^e^ system equipped with a TGA 850 thermobalance was used with the ramp rate of 10 °C min^−1^. Small angle X-ray diffraction patterns were collected on a PANalytical X’Pert PW3710 MPD diffractometer with X-ray sourced from monochromatic CuKα line (λ = 1.54056 Å). The contents of Ti and P were determined on an Agilent microwave plasma-atomic emission spectrometer (MP-AES 4200) after a total dissolution in 65% HNO_3_ and 1% HF using a CEM MARS 5 microwave digestion system. Nitrogen physisorption measurements were carried out on an Autosorb-1 instrument from Quantachrome Instruments. The samples were degassed at 200 °C for 5 h prior to analysis. The BET surface area was determined in the pressure range of 0.05–0.20 P/Po, while the pore size distribution was assessed using the NLDFT model for cylindrical pores.

## Results and discussion

Six ordered mesoporous silica materials with different pore sizes and functional groups were synthesized and characterized with various methods. Then SPME Arrows coated with these materials were fabricated and compared with commercial SPME Arrows for selective extraction of amines at under 1% and 50% humidity levels. The parameters affecting the selectivity of mesoporous silica materials towards LMWAAs were studied. Two SPME Arrow-GC-MS methods were established by using the two most selective mesoporous silica materials for the identification and quantitation of trace amines in mushroom, atmospheric air and urine samples.

### Materials characterization

**Fig. 2:**
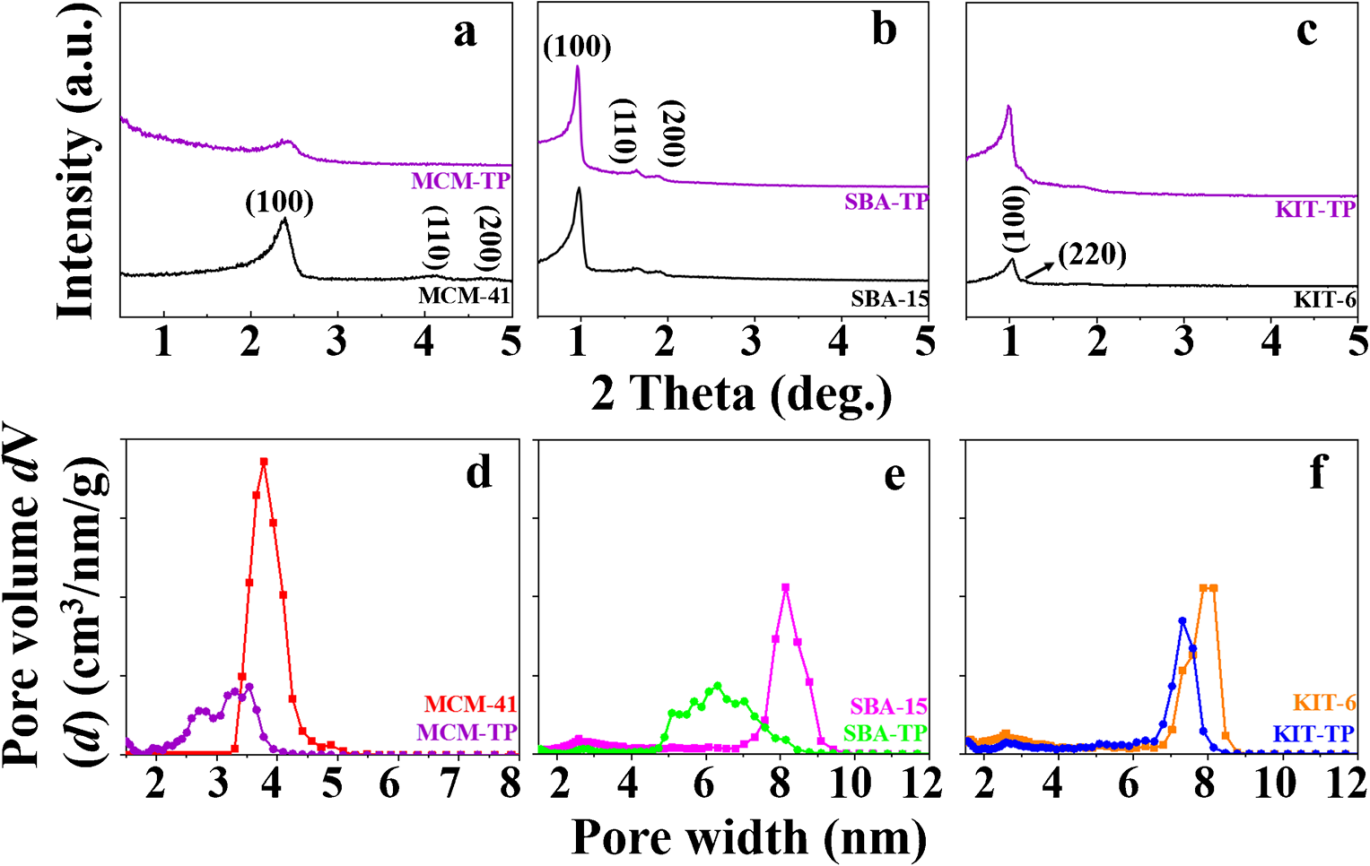
Small-angle XRD patterns (0.5–5°) and density functional theory (DFT) pore size distribution of (**a** and **d**) MCM-41 series, (**b** and **e**) SBA-15 series, and (**c** and **f**) KIT-6 series silica materials

In this work, three types of amorphous mesoporous silica materials were employed as substrates for the synthesis of adsorbents due to their flexible pore sizes and structures. MCM-41 and SBA-15 have two-dimensional (2D) hexagonal pore structures (Fig. 2a-b) with distinctive pore sizes (~4 vs ~8 nm) (Fig. 2d-e) [28, 29], and KIT-6 has a specific 3D cubic structure composed of two intertwined subnetworks (Fig. 2c) and approximately the same pore size as SBA-15 (Fig. 2f) [30]. After grafted with the -TP layer inside the wall, the average pore size and pore volume of silica decreased by ~1 nm and 0.24–0.49 cm^3^ g^−1^, respectively (Fig. 2d-f). The results from small angle XRD (Fig. 2a-c) demonstrated that the (100) reflection of the mesoporous silica decreased after -TP layer modification. This means that the modification resulted in ordered smaller pores, with some pore blockages. The layer-by-layer grafting technique of -TP layer to silica surface, described in literature [31] resulted in reproducible results. The nitrogen sorption-desorption and XRD data (Fig. 2) indicates that the -TP groups are preferentially grafted onto the mesopore walls rather than filling up the pores. Further, the grafted groups, ~8–12 wt.% of -T and ~4–5 wt.% of -P (Table S1), can also enhance the surface acidity of silica materials and thus promote their interaction affinity with basic analytes [32, 33]. Therefore, comparison of the extraction performance of the aforementioned materials can help us to better understand the relation between extraction selectivity and the pore information and/or functional groups.

**Fig. 3:**
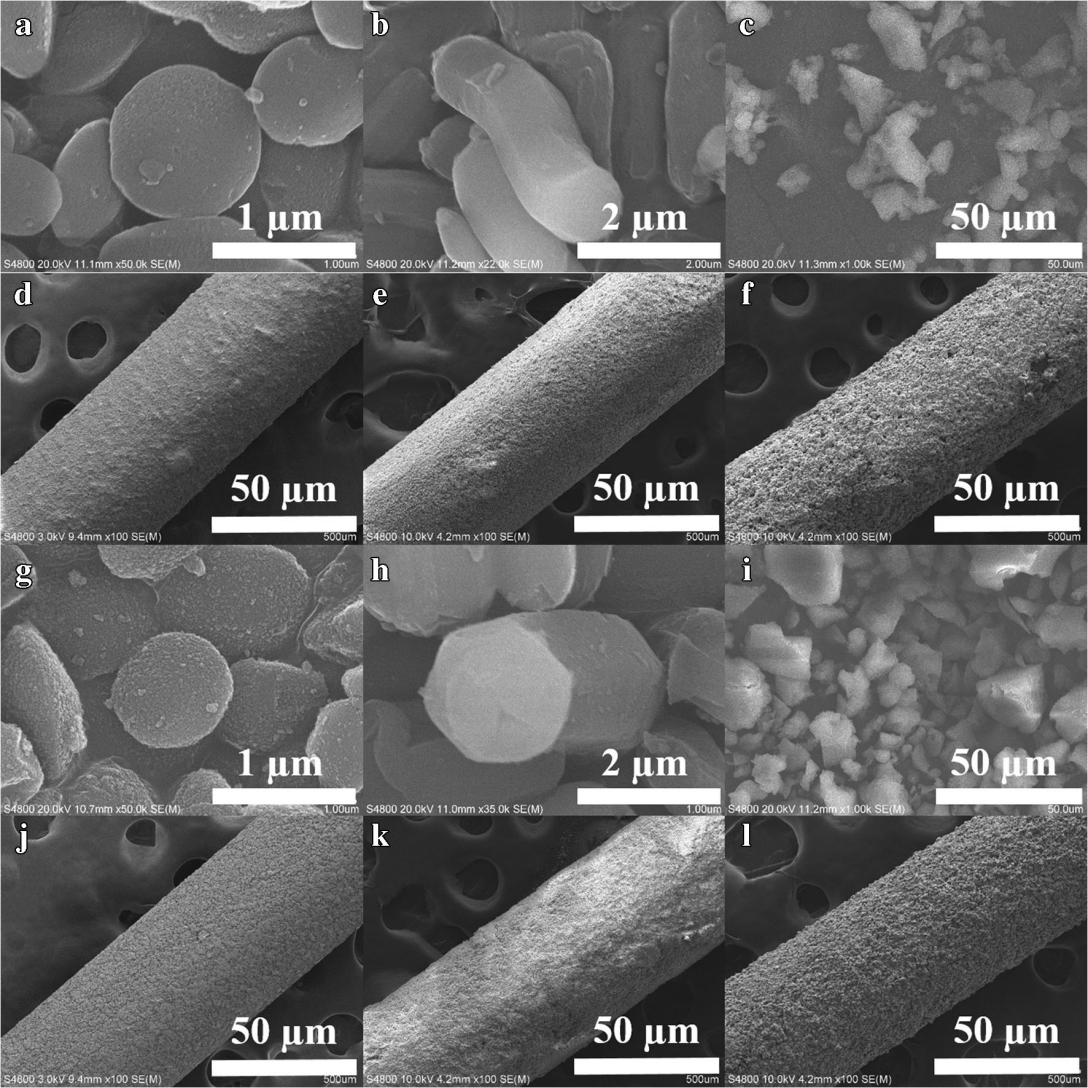
Scanning electron microscopy (SEM) images of (**a**) MCM-41, (**b**) SBA-15, (**c**) KIT-6, (**d**) MCM-TP, (**e**) SBA-TP and (**f**) KIT-TP SPME Arrow coatings (scale bar, 50 μm)

Upon functionalization, the three silica samples preserved their pore architectures based on the small-angle X-ray diffraction (XRD) patterns (Fig. 2a-c). For silica substrates, the weight loss was almost negligible below 600 °C (Figures S2a-c). Even though the thermal stability of silica materials modified by -TP groups slightly decreased, this had no effect on their suitability for thermal desorption, which is normally carried out under 300 °C. Furthermore, their particle morphologies were not altered after functionalization regardless of the spherical MCM-41, cylinder-like SBA-15 or rock-like KIT-6 morphologies (Fig. 3). The corresponding silica-coated SPME Arrows demonstrated good surface uniformity with 20–45 μm coating thickness (insets in Fig. 3a-f). Based on these characterization results, six ordered mesoporous silica materials and their corresponding SPME Arrows were successfully synthesized and fabricated, respectively.

### Selectivity test and selectivity mechanism of SPME Arrows

Since six siliceous adsorbents designed provided selective and rapid extraction of LMWAAs, a standard mixture comprised of EA, DEA, TEA and aniline, representing primary, secondary, tertiary and aromatic amines, was selected to enable a comparison of the extraction affinity between self-made and commercial adsorbents. SPME Arrow with PDMS-DVB coating was the best commercial product for comparison extraction of LMWAAs after a preliminary test (Figure S3).

**Fig. 4:**
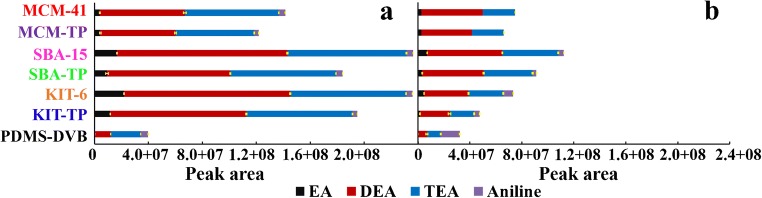
Comparison of tailor-made and commercial SPME Arrows for extraction of EA, DEA, TEA and aniline at (**a**) under 1% and at (**b**) 50% humidity

In order to investigate the extraction affinity of the materials to LMWAAs, SPME Arrows were operated at under 1% humidity to eliminate the interference from moistures. Siliceous coatings extracted 18.6–102.5 times, 4.8–10.8 times and 2.6–4.0 times more EA, DEA and TEA, respectively, than SPME Arrow with PDMS-DVB coating (Fig. 4a). The high extraction efficiencies observed for these six tailor-made coatings are thanks to their excellent porous structure and acidic surface. Pore size is a key factor here especially when the extraction time (10 min) is far from equilibrium (Fig. 6). KIT-6 and SBA-15, with similar pore sizes of 7.9 vs 8.2 nm, extracted approximately the same amounts of LMWAAs even though differences in BET specific surface area (210 m^2^ g^−1^) and pore volume (0.06 cm^3^ g^−1^) (Table S2). On the contrary, MCM-41, with a pore size of 3.8 nm, and the highest surface area of 1182 m^2^ g^−1^ and similar pore volume, resulted only in 60% relative extraction compare to the other two silica materials. The pore size played an important role in the passive SPME sampling due to the random diffusion of molecules into the pore channels. On the other hand, commercial DVB had the largest pore size of 40 nm [34] but lowest extraction capacity of LMWAAs among the materials. This revealed that surface functional groups are also key factors for LMWAAs capturing. DVB with a more neutral and hydrophobic surface, has limited interactions with highly polar LMWAAs. However, it has good π-π interactions with aromatic aniline. As well recognized silica substrates have strong interactions with basic analytes due to their surface silanol groups. The surface Lewis and Brønsted acidity was further enhanced by modification with -TP groups. However, surface grafting decreased the pore sizes and volumes of the materials. The pore sizes of MCM-41, SBA-15 and KIT-6 dropped by ~0.3, ~1.8 and ~0.5 nm, respectively, due to the -TP modification (Fig. 2d-f). Accordingly, this process reduced also the amount of the extracted LMWAAs by 13.9, 22.1 and 17.3%, which again supported the importance of pore size in capturing small molecules (Fig. 4a).

For further evaluation, each SPME Arrow was processed under 50% humidity because of the unignorable moisture in most natural samples. Thus, this experimental design is more valuable for investigating their applicability in natural samples. As expected, the extraction efficiency of SPME Arrow with siliceous coatings to LMWAAs decreased dramatically at a higher humidity due to their hydrophilic surface which can be occupied by water instead of target molecules (Figure S4). Among the silica materials, SPME Arrow with KIT-6 and KIT-TP coatings provided biggest extraction drops of 71 and 77%, respectively (Fig. 4b), because of their 3D cubic pore structure and 6–8 nm pore size, which are the most accessible pores for water molecules. Interestingly, the 2D structure and ~3 nm channels of the MCM-41 and MCM-TP materials did not allow most water molecules (Figure S4) to enter their pores, giving the smallest decrease of 44 and 45% in the extraction (Fig. 4b). The hydrophobic composition of SPME Arrow with PDMS-DVB decreased the extraction with 49%, proving that it is easy for water molecules to enter its 40 nm pores (Fig. 4b). Despite of the drop in extraction performance of silica materials, their extraction efficiency to EA, DEA and TEA was still 11.6–53.3, 3.3–8.4 and 1.8–3.6 times higher than that of commercial SPME Arrow with PDMS-DVB. The results indicate that pore size is not only an important parameter for controlling LMWAAs extraction but also for preventing the interferences caused by water.

A viable extraction strategy of LMWAAs from complicated matrices does not only need a high adsorbent capacity but also selectivity, efficiency and recyclability play an important role.

**Fig. 5:**
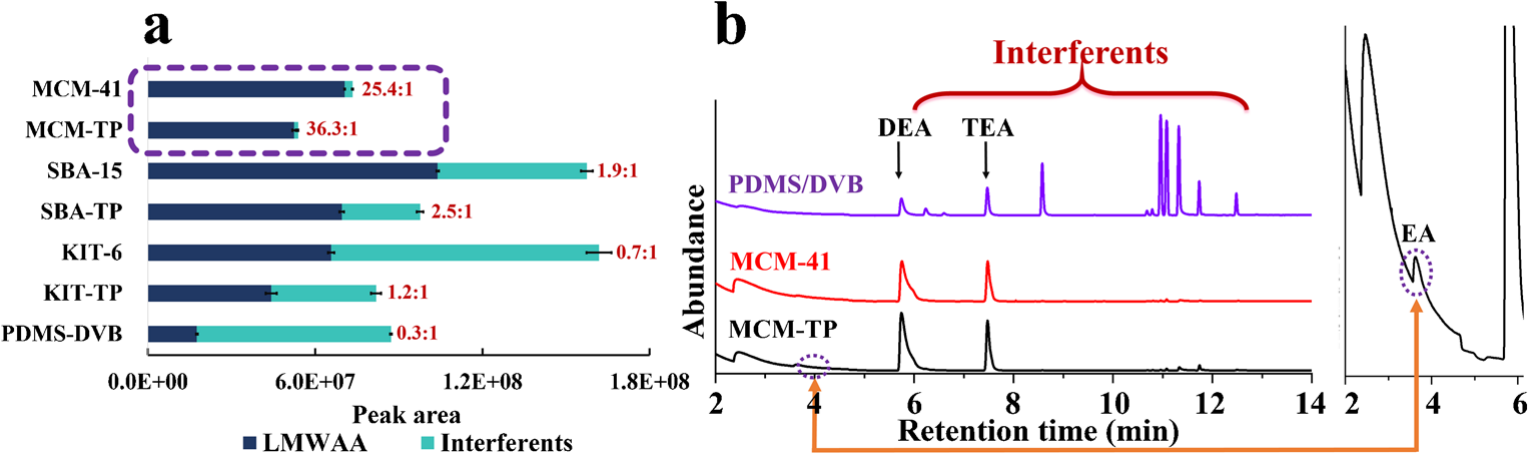
**a** Selectivity comparison of different materials towards LMWAAs and **b** represent GC-MS chromatograms of LMWAAs and other compounds in samples extracted with MCM-41, MCM-TP and PDMS-DVB-SPME Arrows

The laboratory-prepared SPME Arrow silica and SPME Arrow PDMS-DVB coatings were then evaluated in terms of their ability to extract LMWAAs from a mixture containing ten representative volatile organic compounds (VOCs) ranging from 0.07 to 12.58 ng mL^−1^ of aliphatic and aromatic compounds. They included ketones, alcohols, furans, hydrocarbons and acetates (Table S3). For this mixture, SPME Arrow with MCM-41 and MCM-TP coatings gave the highest selectivity factor (defined as: total peak area ratio of LMWAAs/ten competing compounds) of 25.4:1 and 36.3:1, respectively (Fig. 5a). This can be explained by the synergism of surface functionality, pore size and pore structure of these two materials. After functionalized with the -TP group, silica materials enhanced their affinity to amines, and at the same time, their small pore size restricted the diffusion of low affinity interferents. On the other hand, due to the largest pore size and most accessible 3D pore structure, SPME Arrow with KIT-6 series therefore logically had the lowest selectivity to the amines. Although SPME Arrow with SBA-15 series had approximately the same pore size, their 2D channels prevents molecules to diffuse in, giving slightly higher selectivity factor while still much lower than that of SPME Arrow MCM-41 series. As an example, the chromatogram in Fig. 5b clearly shows the superiority and weak selectivity of SPME Arrow MCM-41 series and PDMS-DVB coatings, respectively. In summary, SPME Arrow MCM-41 series silica had the lowest capacity but the best selectivity to LWMAAs because of 1) their 2–4 nm pore sizes capable to capture small molecules, 2) their acidic surface that has strong affinity to LMWAAs, 3) their 2D pore channels that limit the extraction of low affinity target compounds and water molecules, and 4) their nonaromatic structure that has low affinity to aromatic compounds. Moreover, SPME Arrow with MCM-41 series coatings should also work for selective extraction of other LMWAAs, such as methylamine, dimethylamine, trimethylamine, etc.

**Fig. 6:**
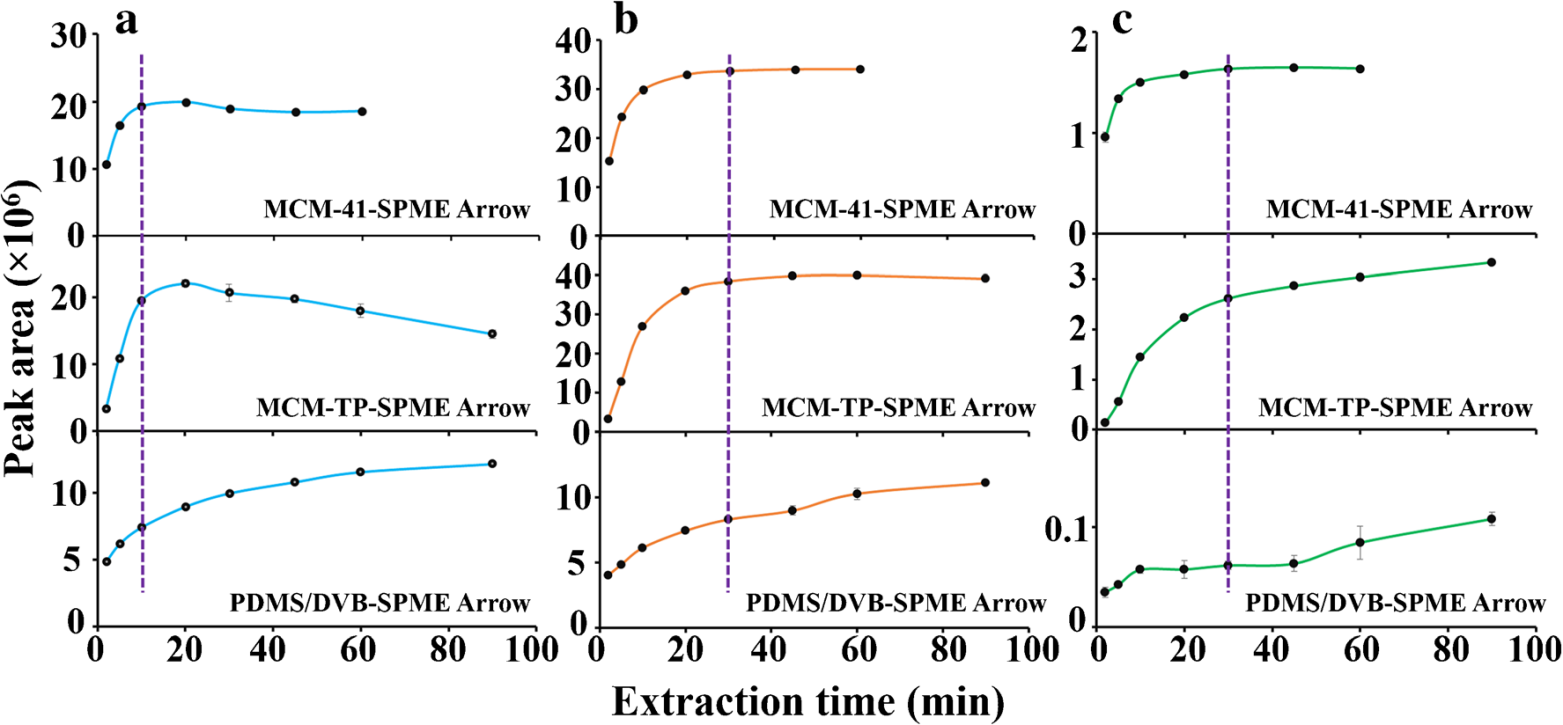
Extraction kinetics of MCM-41 series and PDMS-DVB SPME Arrows for extraction of (**a**) TEA, (**b**) DEA and (**c**) EA

In addition, MCM-41- and MCM-TP-SPME Arrows reached extraction equilibrium for DEA and TEA within 20 and 30 min, respectively, which were much shorter than that of SPME Arrow with PDMS-DVB (60 min) (Fig. 6). This remarkable, fast extraction rate is attributed to the acidic surface groups which strongly trapped the analytes. However, MCM-TP-SPME Arrow extracted more EA with longer extraction time. Obviously, there is a competition in the extraction of EA and TEA by SPME Arrow with MCM-TP coating after 10 min, indicating that the material’s acidic channels prefer to interact with smaller and more polar EA. As a compromise of the extraction of EA and TEA, the extraction time should not exceed 30 min by MCM-TP-SPME Arrow.

To study the repeatability of extraction of LMWAAs by MCM-41- and MCM-TP-SPME, more than 150 extraction cycles were carried out. Since MCM-41- and MCM-TP-SPME Arrows gave repeatable results, they were selected for the further studies.

### Optimization of mesoporous silica-coated SPME Arrow conditions

The following parameters, which can influence the SPME Arrow performance, were optimized: (a) base addition; (b) ionic strength; (c) extraction temperature; (d) agitation speed; (e) desorption temperature and (f) desorption time. Respective data and Figures are given in the Supporting Material (Figure S5). For the optimization studies a 100 ng mL^−1^ mixture of EA, DEA and TEA was used.

#### Extraction conditions

The addition of base to the amine solution is crucial to neutralize and release the amines into the headspace.

During the extraction, higher temperature usually promotes the transfer of analytes from the solution to the headspace, but it can also increase the amount of water and its condensation on the SPME Arrow [35]. In addition, analytes already attached on the sorbent can be partitioned back to the headspace (equilibrium favors the gas phase) due to the high temperature. Three temperatures, 40, 50 and 60 °C, were employed after considering the temperature limit of the PAL auto-sampler and water condensation. The lowest temperature, 40 °C, gave the strongest response which revealed an exothermic process. Also, the effect of different agitation speeds, 250, 500, 750, and 1000 rpm, on the recovery was tested with two SPME Arrow systems without observing any clear trend.

#### Desorption conditions

The amount of desorbed analytes greatly increased with higher desorption temperature and longer desorption times. However, a compromise between these two parameters was made to guarantee a long coating lifetime, while still maintaining an efficient desorption. A 60 s desorption step at 260 °C ensured over 95% release of amines from the two coatings tested.

In summary, the extraction and desorption conditions for the amines with MCM-41- and MCM-TP-SPME Arrows were generally the same: (a) 250 μL 5 M KOH; (b) 2 g NaCl; (c) 40 °C extraction temperature; (d) 250 rpm agitation speed; (e) 260 °C desorption temperature and (f) 60 s desorption time. Extraction time was exceptionally 20 and 30 min for MCM-41- and MCM-TP-SPME Arrows, respectively.

### Method validation

The performance of the MCM-41- and MCM-TP-SPME Arrows for amine analysis under the optimized conditions (calibration plots, limits of detection (LODs), limits of quantitation (LOQs), coefficient of determination and repeatability) is shown in Table S4. Triplicate measurements at eight concentration levels were processed for the calibration plots. LODs and LOQs were determined as concentrations giving the signal-to-noise ratio of three and ten, respectively. In general, MCM-41-SPME Arrow-GC-MS exhibited slightly lower LODs and LOQs than that of MCM-TP-SPME Arrow-GC-MS because of its higher extraction capacity. Both materials showed linearity with good correlation coefficients (*R*^2^), which were higher than 0.9955, and good repeatability (RSD < 20.1%). The method sensitivity for TEA determination in this study was two magnitudes higher than that in our previous work [14]. On the other hand, MCM-41 and MCM-TP-SPME Arrow-GC-MS methods demonstrated advantages over to other SPME-based methods for LMWAA determinations in terms of selectivity, sensitivity, linear range or repeatability (Table S5).

### Sample analysis

#### Mushroom samples

In the six mushroom samples, DEA and TEA were detected and their concentrations ranged from 181.7 ± 6.1 to 373.3 ± 19.2 and 18.4 ± 2.1 to 41.8 ± 0.7 ng g^−1^ by MCM-41-SPME Arrow and 174.9 ± 1.0 to 375.7 ± 12.4 and 19.5 ± 2.5 to 38.5 ± 2.6 ng g^−1^ by MCM-TP-SPME Arrow, respectively. The results proved also that there was a good correlation between two different self-made SPME Arrows. Mushroom sample #2 was used for recovery test and it was spiked with three amines at 20, 50 and 100 ng g^−1^ levels. Both MCM-41- and MCM-TP-SPME Arrows gave good recoveries (≥75.9%) and repeatability (≤14.5%) (Table 1).

**Table 1:** Analytical results for mushroom samples by MCM-41- and MCM-TP-SPME Arrow extraction and GC-MS analysis

Mushroom	MCM-41-SPME Arrow			MCM-TP-SPME Arrow		
	EA (ng g^−1^)	DEA (ng g^−1^)	TEA (ng g^−1^)	EA (ng g^−1^)	DEA (ng g^−1^)	TEA (ng g^−1^)
#1	ND	288.9 ± 4.3	23.5 ± 0.8	ND	308.4 ± 12.9	30.4 ± 2.3
#2	ND	334.5 ± 6.8	34.6 ± 4.2	ND	354.6 ± 12.3	34.3 ± 0.4
#3	ND	282.3 ± 7.1	41.8 ± 0.7	ND	291.9 ± 23.1	38.5 ± 2.6
#4	ND	260.0 ± 15.1	28.7 ± 0.7	ND	250.5 ± 3.7	28.8 ± 0.8
#5	ND	181.7 ± 6.1	18.4 ± 2.1	ND	174.9 ± 1.0	19.5 ± 2.5
#6	ND	373.3 ± 19.2	24.1 ± 1.7	ND	375.7 ± 12.4	27.1 ± 1.6
#2	Recovery (RSD) %	Recovery (RSD) %	Recovery (RSD) %	Recovery (RSD) %	Recovery (RSD) %	Recovery (RSD) %
20 ng g^−1^	81.1 (7.9)	93.6 (7.5)	83.3 (4.3)	75.9 (2.1)	97.1 (14.5)	87.8 (4.6)
50 ng g^−1^	91.1 (3.8)	95.6 (6.5)	89.6 (12.0)	86.6 (11.5)	89.9 (1.7)	95.4 (4.3)
100 ng g^−1^	104.1 (2.6)	91.6 (1.5)	102.2 (8.9)	96.6 (7.5)	86.6 (5.8)	105.1 (1.0)

#### Atmospheric air samples

MCM-TP-SPME Arrow was used for the analysis of atmospheric air samples. DEA concentration ranged between 6.2–103.7 ng m^−3^ in the first three days and TEA concentration ranged between 0.1–8.6 ng m^−3^ in four days (Figure S6). The results revealed that there were amine emissions in the boreal forest even at low temperatures (−1.4–3.3 °C) and the snow in the last two days decreased their levels significantly. Amine concentrations in this study were slightly higher but still in the same order of magnitude as those reported in the literature [4, 5].

#### Urine sample

**Fig. 7:**
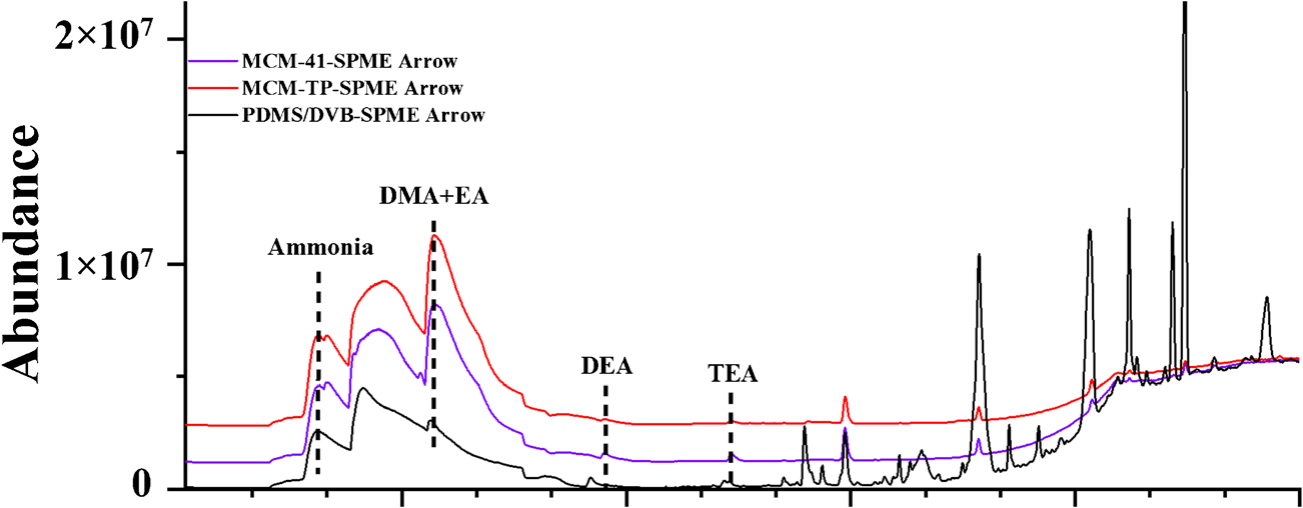
GC-MS chromatograms of the urine samples after extraction by MCM-41, MCM-TP and PDMS-DVB coated SPME Arrows

The urine sample was analyzed by GC-MS utilizing MCM-41-, MCM-TP- and PDMS-DVB-SPME Arrow systems (Fig. 7). Laboratory-made SPME Arrows gave much more intense amine peaks and clearer baselines compared to the commercial PDMS-DVB-SPME Arrow. In the sample, ammonia, dimethylamine (DMA) + EA, DEA and TEA were identified with all laboratory-made SPME Arrows mentioned above.

## Conclusions

In this study, SPME Arrows including different self-made mesoporous silica coatings were utilized to capture rapidly targeted LMWAAs from various sample matrices. SPME Arrows with MCM-41 and MCM-TP coatings having 2D channels and a pore size of <4 nm gave superior selectivity towards LMWAAs when compared to another self-made SPME Arrow silica coatings SBA-15 and KIT-6, and to several commercial materials. The success of the extraction and especially the extraction selectivity by using different SPME Arrow coatings were studied by clarifying two main properties: 1) size exclusion, in terms of pore size and pore structure, and 2) acid-base interaction, by increasing silica surface acidity by functionalization with -TP groups resulting in more acidic Lewis and Brønsted sites. Furthermore, optimal laboratory-made SPME Arrows demonstrated fast extraction kinetics, good reusability and convenience for the development of analytical methods. The SPME Arrow –GC-MS methods at optimal conditions were validated and further applied to the identification and quantitation of LMWAAs in mushroom, atmospheric air and urine samples. The results achieved indicated that functionalized mesoporous silica was suitable for the extraction and enrichment of LMWAAs in different sample matrices. With a careful selection of silica substrate and functional groups, new materials allow to capture selectively many other small and polar VOCs from liquid or gaseous samples. Furthermore, an exhaustive sampling technique, such as in-tube extraction and needle trap extraction, can improve the method sensitivity and the quantitation further.

## Electronic supplementary material

ESM 1 (DOCX 2.39 MB)
