# Supplementary material for: Selective extraction of aliphatic amines by functionalized mesoporous silica-coated solid phase microextraction Arrow
Source: Mikrochim Acta. 2019 Jun 11;186(7):412. doi: 10.1007/s00604-019-3523-5 (PMC6560004; doi:10.1007/s00604-019-3523-5)
Supplement: Supplementary file 1 — (DOCX 2.39 MB) [file 604_2019_3523_MOESM1_ESM.docx]

**Electronic Supporting Material on the Microchimica Acta publication entitled**

**Selective extraction of aliphatic amines by functionalized mesoporous silica-coated solid phase microextraction Arrow**

**Hangzhen Lan,^a,b^ Wenzhong Zhang,^a^ Jan-Henrik Smått,^c^ Risto T. Koivula,^a^ Kari Hartonen^a,b^ and Marja-Liisa Riekkola^a,b,^**

**^a^ Department of Chemistry, P.O. Box 55, 00014 University of Helsinki, Finland**

**^b^ Institute for Atmospheric and Earth System Research, P.O. Box 64, 00014 University of Helsinki, Finland**

**^c^Laboratory of Physical Chemistry, Åbo Akademi University, Porthansgatan 3-5, 20500 Turku, Finland**

**Abstract**

Mesoporous silica-coated solid phase microextraction (SPME) Arrow systems were developed for capturing of low-molecular-weight aliphatic amines (LMWAAs) from various complicated sample matrices. Specifically, silicas of type MCM-41, SBA-15 and KIT-6 were chosen as substrates to afford size-exclusion selectivity. They possessed ordered multidimensional pore-channel structures and mesopore sizes between 3.8 and 8.2 nm. Their surface acidity was enhanced by grafting them with a layer of titanium hydrogenphosphate (-TP). This enhanced the chemical selectivity for basic LMWAAs. The siliceous coatings increased the extraction of ethylamine, diethylamine (DEA) and triethylamine (TEA) by factors of 18.6-102.5, 4.8-10.8 and 2.6-4.0, respectively, when compared to the commercial SPME Arrow with polydimethylsiloxane/divinylbenzene coating. Among them, the MCM-41 and MCM-41-TP coated SPME Arrows demonstrated exceptional selectivity towards LMWAAs that were quantified by gas chromatography-mass spectrometry (GC-MS). The total peak area ratios of LMWAAs/ten competing compounds were 25.4 and 36.3, respectively. The extraction equilibrium was reached within 20-30 min. The MCM-41 and MCM-41-TP derived SPME Arrows gave very similar results (18.4±2.1–376±12 ng g^-1^ to DEA and TEA) when applied to urban mushroom samples. SPME Arrow with MCM-41 coatings followed by GC-MS was applied also to the analysis of atmospheric air and urine samples resulting in high selectivity due to the size and mesoporous structure of the functionalized silica, and its chemical interactions with the LMWAAs.

**Materials synthesis**

The syntheses of mesoporous MCM-41, SBA-15 and KIT-6 silica materials were performed in batches of 10 g scale and the subsequent calcination of the obtained materials at 550 °C removed the organic templates.

The functionalization of the mesoporous silica with titanium phosphate moieties was conducted as follow: briefly, 1 g of silica material was placed into a two-neck flask sealed with a rubber septum and connected to a standard argon Schlenk line. After substituting air with argon in the flask, 20 mL of dry toluene was added through the septum. The slurry was stirred at room temperature for 1 h, after which 3.4 mmol of Ti(OPr*^i^*)_4_ was added. The reaction mixture was gradually heated to 80 °C and refluxed for 2 h. The product, after cooling, was successively washed with 20 mL of toluene (three times) and 20 mL of water (three times) to eliminate the unreacted Ti precursor and to hydrolyze the alkoxy groups. Drying of the obtained solid in air at 80 °C gave the titanium-modified mesoporous silica materials. The functionalization of the phosphate groups was done by treating the titanium-modified materials with the same procedure as used for modification with titanium, except that 10.2 mmol of the phosphorus precursor POCl_3_ was added instead of the Ti precursor.


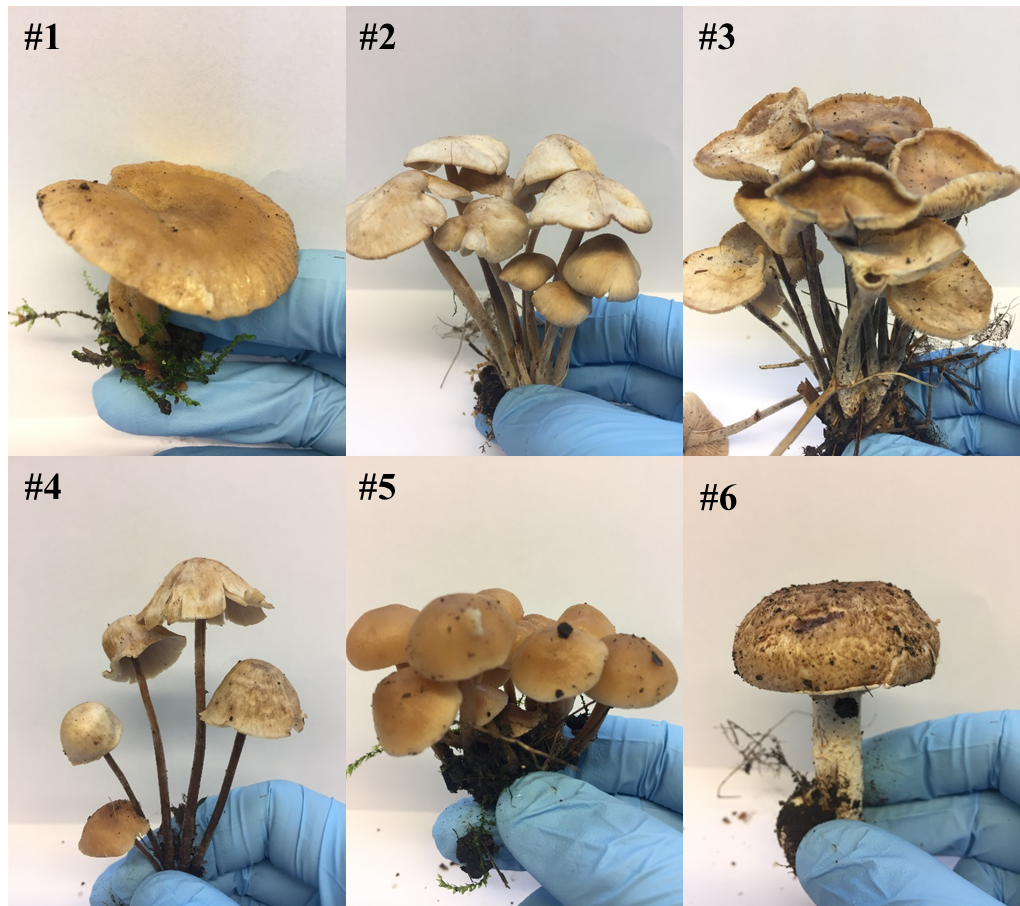


Figure S1. Six types of mushroom, collected near Kumpula Campus, University of Helsinki, Helsinki, Finland, were used in this study.


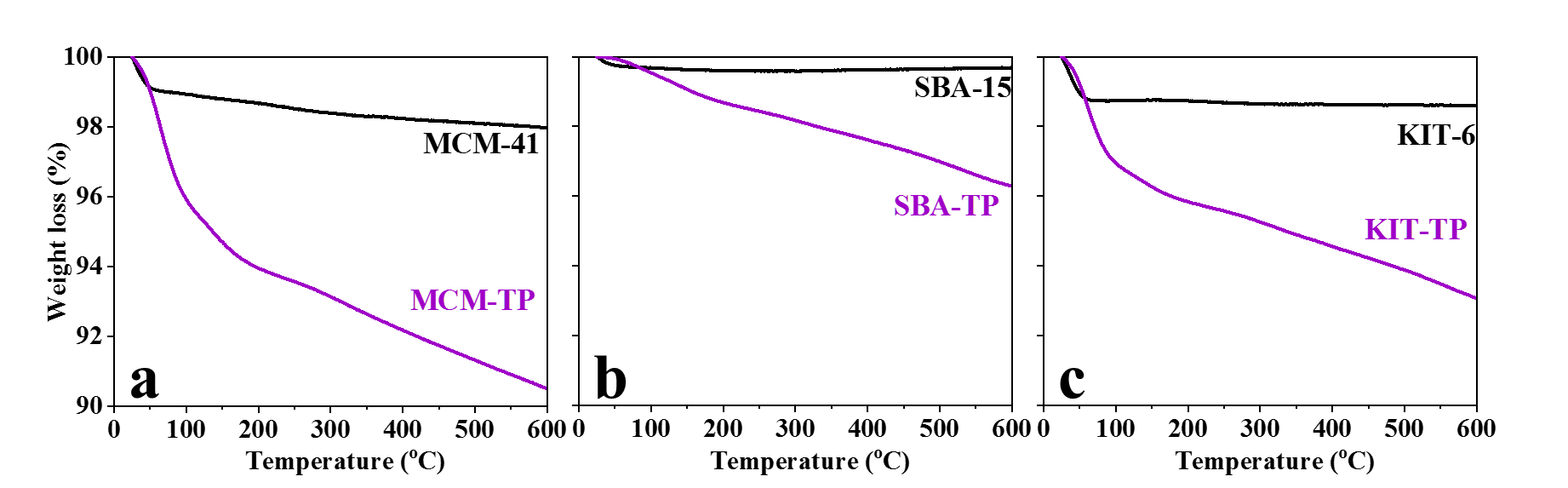


Figure S2. TGA patterns (~20-600 °C) of a) MCM-41 series, b) SBA-15 series, and c) KIT-6 series silica materials.


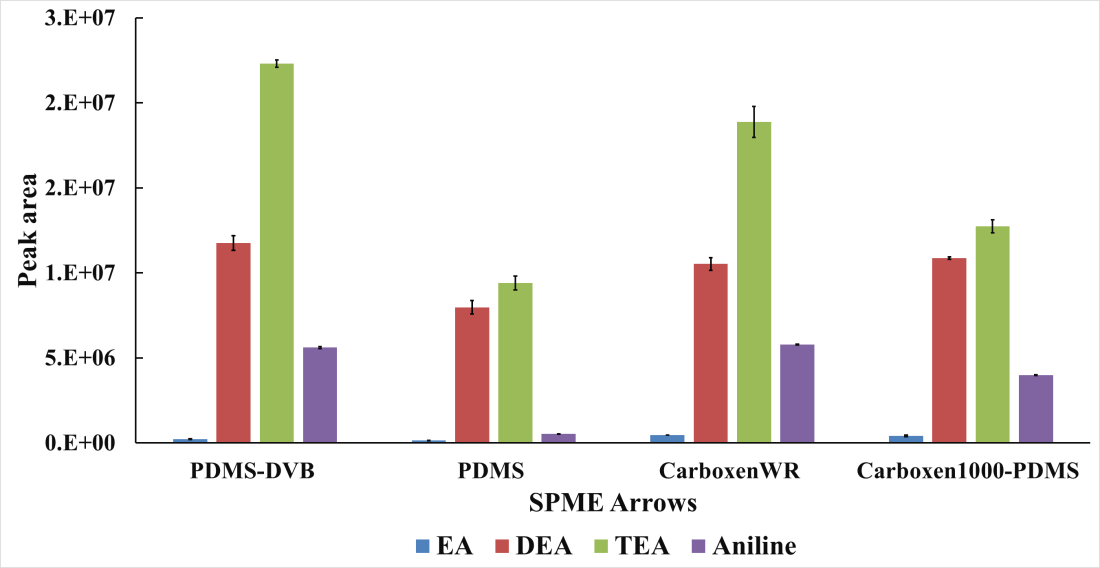


Figure S3. Comparison of commercial SPME Arrows for extraction of ethylamine (EA), diethylamine (DEA), triethylamine (TEA) and aniline.


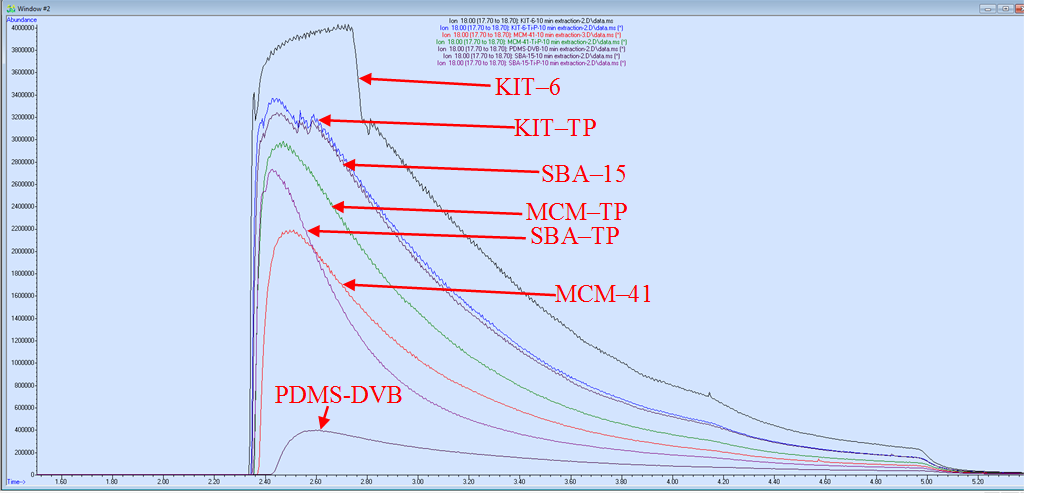


Figure S4. Selected ion chromatograms (*m/z*=18) of water, extracted by seven different SPME Arrow systems at 50% humidity.


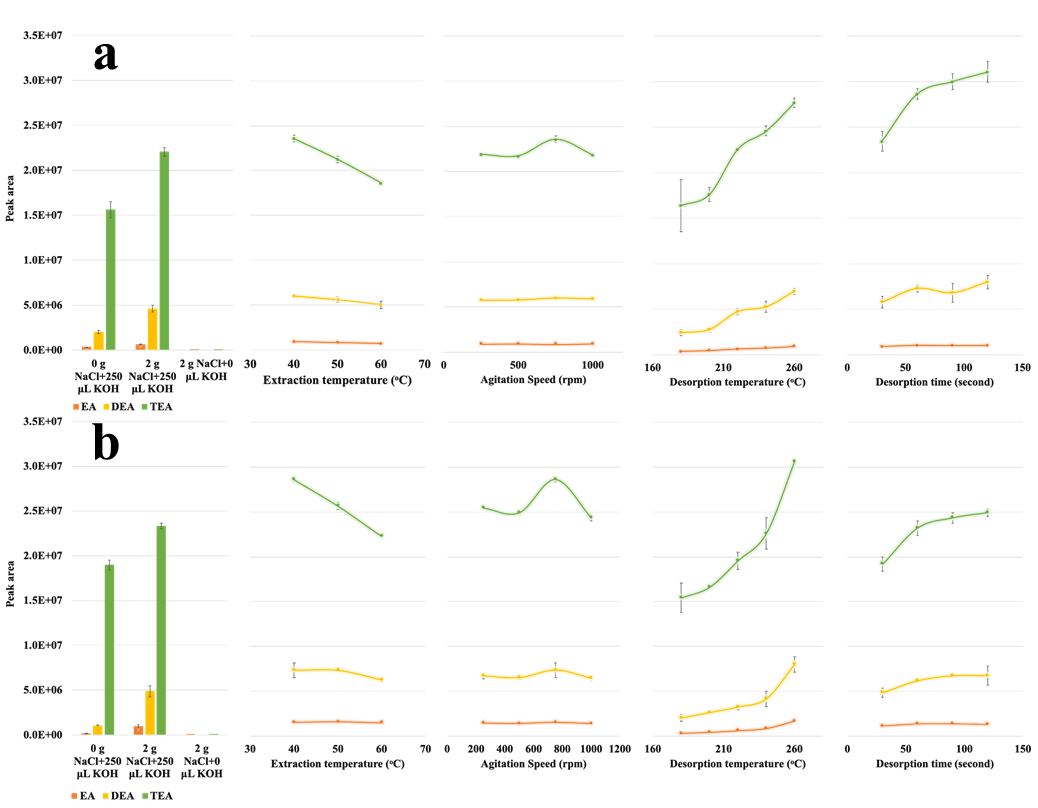


Figure S5. Optimization of extraction and desorption conditions of a) MCM-41 and b) MCM-TP coated SPME Arrows.


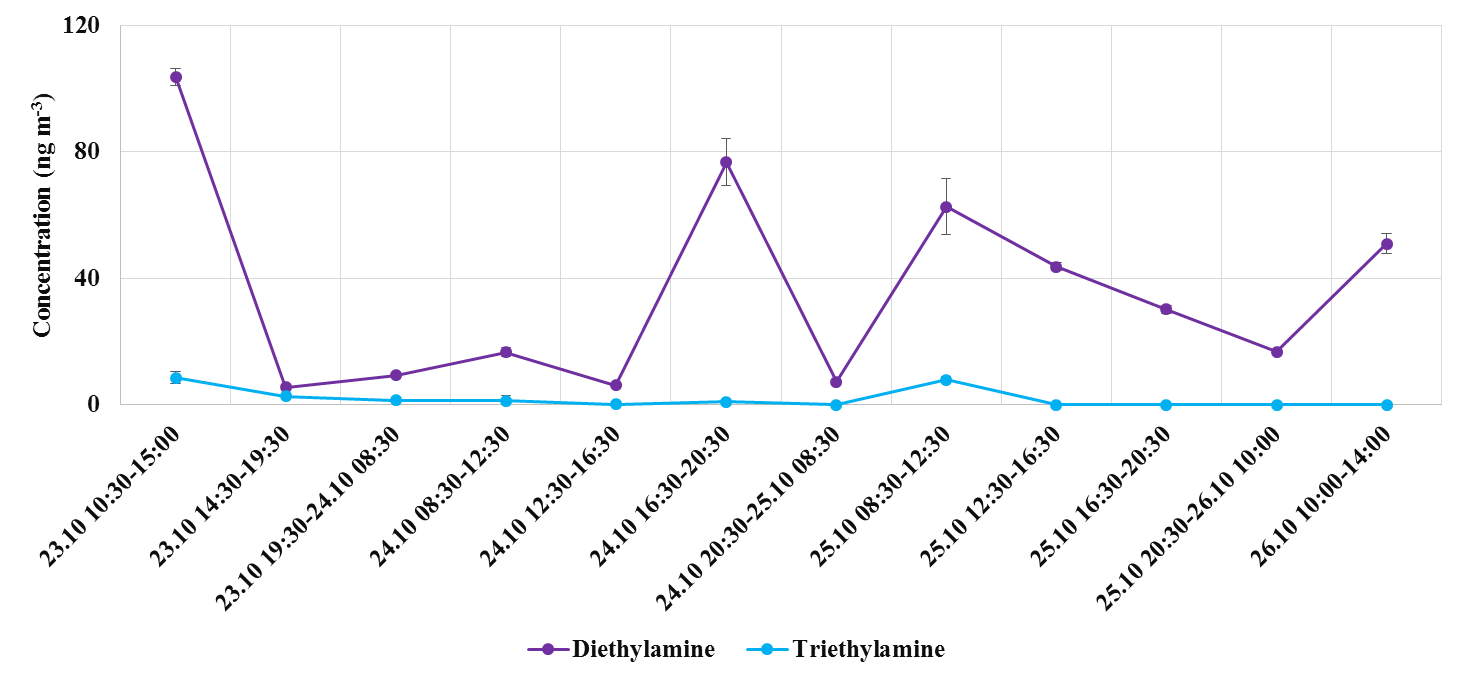


Figure S6. Variations of the concentrations of DEA and TEA at SMEAR II Station, Hyytiälä, Finland from 23rd to 26^th^ of October, 2018.

Table S1. Contents of titanium (T) and phosphate (P) in MCM-TP, SBA-TP and KIT-TP.

| Sample | T wt.% | P wt.% |
| --- | --- | --- |
| MCM-TP | 7.71±0.37 | 4.33±0.02 |
| SBA-TP | 8.63±0.13 | 4.76±0.15 |
| KIT-TP | 11.54±0.09 | 5.74±0.09 |

|  | **BET surface area (m²/g)** | **Pore volume* (mL/g)** |
| --- | --- | --- |
| **MCM-41** | 1182 | 0.97 |
| **SBA-15** | 759 | 1.05 |
| **KIT-6** | 969 | 1.11 |
| **MCM-T-P** | 779 | 0.47 |
| **SBA-T-P** | 640 | 0.81 |
| **KIT-T-P** | 704 | 0.78 |

Table S2. BET surface area and pore volume of six silica materials.

*At P/P_o_ = 0.98

Table S3. Concentrations of analytes for selectivity test at 50% humidity.

^a^: n-Octanol/Water partition coefficient.

Table S4. Analytical performance of MCM-41- and MCM-TP-SPME Arrow-GC-MS methods.

|  | Analytes | LODs  (ng mL^-1^) | LOQs  (ng mL^-1^) | Linear range  (ng mL^-1^) | Coefficient of determination  (R^2)^ | RSD  (%) |
| --- | --- | --- | --- | --- | --- | --- |
| MCM-41-SPME Arrow | EA | 2 | 5 | 5-500 | 0.9984 | 0.5-20.1 |
|  | DEA | 0.2 | 1 | 1-200 | 0.9982 | 1.6-9.1 |
|  | TEA | 0.01 | 0.03 | 0.03-200 | 0.9980 | 0.5-14.3 |
| MCM-TP-SPME Arrow |  |  |  |  |  |  |
|  | EA | 3 | 10 | 10-1000 | 0.9997 | 0.7-17.4 |
|  | DEA | 0.8 | 2 | 2-200 | 0.9955 | 0.7-5.8 |
|  | TEA | 0.01 | 0.03 | 0.03-200 | 0.9997 | 0.2-3.6 |

Table 5. Comparison with other sample preparation methods for the determination of LMWAAs.

| Method | Selectivity | Analyte | Sample | LOQ (ng mL^-1^) | Linear range (ng mL^-1^) | Repeatability (RSD %) | Reference |
| --- | --- | --- | --- | --- | --- | --- | --- |
| Derivatized PDMS/DVB-  SPME-GC-MS | Yes | EA | Atmospheric air | 1.72 and 25.9 | 1.7-34.5 and 25.9-86.2 | 9-49 and 11-25 | [1] |
| Acidified zeolitic imidazolate  framework-8-SPME Arrow-GC-MS | Yes | TEA | Wastewater,  mushroom,  and salmon | 1 | 1-500 | 2.6-10.1 | [2] |
| Derivatized Polyacrylate-  SPME-GC-MS/MS | Yes | EA | Wastewater | 0.05 | 0.05-17.5 | 5 | [3] |
| Pressurized hot water extraction-  derivatized Polyacrylate-  SPME-GC-MS/MS | Yes | EA | Sewage sludge | 150 ng g^-1^ | 150-10000 ng g^-1^ | 7 | [4] |
| Derivatized Polyacrylate-  SPME-GC-MS | Not mentioned | DEA | Wine | 0.428 | 1-1000 | 3.5-10 | [5] |
| This article | Yes | EA, DEA and TEA | Mushroom,  atmospheric air,  and urine | 5-10 (EA)  1-2 (DEA)  0.03 (TEA) | 5-500 and 10-1000 (EA)  1-200 and 2-200 (DEA)  0.03-200 (TEA) | 0.5-20.1 and 0.7-17.4 (EA)  1.6-9.1 and 0.7-5.8 (DEA)  0.5-14.3 and 0.2-3.6 (TEA) |  |

References

[1] Parshintsev J, Rönkkö T, Helin A, Hartonen K, Riekkola M-L (2015) Determination of atmospheric amines by on-fiber derivatization solid-phase microextraction with 2, 3, 4, 5, 6-pentafluorobenzyl chloroformate and 9-fluorenylmethoxycarbonyl chloride. Journal of Chromatography A 1376:46-52

[2] Lan H, Rönkkö T, Parshintsev J, Hartonen K, Gan N, Sakeye M, Sarfraz J, Riekkola M-L (2017) Modified zeolitic imidazolate framework-8 as solid-phase microextraction Arrow coating for sampling of amines in wastewater and food samples followed by gas chromatography-mass spectrometry. Journal of Chromatography A 1486:76-85

[3] Llop A, Pocurull E, Borrull F (2010) Automated determination of aliphatic primary amines in wastewater by simultaneous derivatization and headspace solid-phase microextraction followed by gas chromatography–tandem mass spectrometry. Journal of Chromatography A 1217(4):575-581

[4] Llop A, Borrull F, Pocurull E (2010) Pressurised hot water extraction followed by simultaneous derivatization and headspace solid-phase microextraction and gas chromatography-tandem mass spectrometry for the determination of aliphatic primary amines in sewage sludge. Analytica Chimica Acta 665(2):231-236

[5] Papageorgiou M, Lambropoulou D, Morrison C, Namieśnik J, Płotka-Wasylka J (2018) Direct solid phase microextraction combined with gas chromatography–Mass spectrometry for the determination of biogenic amines in wine. Talanta 183:276-282
